# Evaluation of antioxidant properties of nanoencapsulated sage (*Salvia officinalis* L.) extract in biopolymer coating based on whey protein isolate and Qodumeh Shahri (*Lepidium perfoliatum*) seed gum to increase the oxidative stability of sunflower oil

**DOI:** 10.1002/fsn3.3177

**Published:** 2022-12-12

**Authors:** Behnaz Safarpour, Reza E. Kenari, Jamshid Farmani

**Affiliations:** ^1^ Department of Food Science and Technology Sari Agricultural Sciences and Natural Resources University Sari Iran

**Keywords:** emulsion, natural extract, oxidation, phenolic compounds

## Abstract

Sage leaf extract (SLE) is considered an excellent source of bioactive compounds mainly because of its high content of phenolics, widely known as natural antioxidants. This study aimed to compare the performance of free/encapsulated SLE by different coatings in protecting sunflower oil against oxidative deterioration. The coating materials were whey protein isolate and qodumeh seed gum at different ratios (1:0, 1:1, and 0:1). Each nanocapsule was analyzed for particle size, zeta potential, encapsulation efficiency, phenolics release, and SEM images. The total phenolic compounds of SLE were 31.12 mg GA/g. The antioxidant activity of SLE was increased in both DPPH and FRAP assays by increasing extract concentration from 50 to 250 ppm. All nanoparticles exhibited nanometric size, negative zeta potential, encapsulation efficiency higher than 60%, and gradual release during storage. The oxidative stability of sunflower oil with or without the incorporation of 250 ppm of free/encapsulated SLE was evaluated during 24 days of storage at 60°C. Peroxide value (PV), thiobarbituric acid value (TBA), oxidative stability index (OSI), color index (CI), and conjugated dienes (CD) were determined. COPM nanoparticles showed the lowest PV, TBA, CI, and CD but both SGUM and WHEY were more effective in delaying oil oxidation than TBHQ and free extract. Higher OSI was observed in oil‐containing nanoparticles with composite coating. Results obtained reinforce the use of whey protein isolate and qodumeh seed gum as a coating for encapsulating SLE to increase the shelf life of sunflower oil as a natural antioxidant.

## Graphical abstract

Nano‐encapsulation is one of the very useful methods for preserving antioxidant compounds, which leads to the controlled release of antioxidant compounds in oil. In our research, Nano‐encapsulation was very effective in preserving and controlling the release of sage extract.

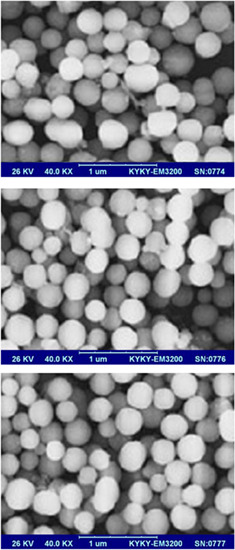

## 1 INTRODUCTION

Sunflower (*Helianthus annus*) is one of the most important oil crops grown worldwide due to high‐yield oil, and lack of antinutritional factors (Aly et al., 2021; Jafari et al., 2022). Sunflower oil (SFO) is a kind of nutritious vegetable oil that contains more than 85% polyunsaturated fatty acids (PUFA), especially linoleic acid which is used for medical treatment (Meng et al., 2021; Sayyari & Farahmandfar, 2017). The ratio of omega‐3 and omega‐6 fatty acids is prominent for providing cardiovascular and heart health benefits (Aly et al., 2021).

However, due to its fatty acid composition with high PUFA, it is one of the most susceptible to suffering rancidity and oxidation progress. Fat oxidation results in unpleasant flavors, discoloration, changes in texture, nutritional value, shelf life, and appearance of SFO, so synthetic antioxidants such as tert‐butyl hydroquinone (TBHQ), propyl gallate (PG), butylated hydroxytoluene (BHT), and butylated hydroxy anisole (BHA) were used (Razavi & Kenari, 2021). Although synthetic antioxidants are attractive due to their low cost, wide availability, great stability, and effectiveness, their use is limited as they may generate health risks, gastrointestinal tract problem, and cancer risk (Xu et al., 2021). Today, there is growing interest to explore natural antioxidants like plant extracts which provide higher antioxidant activity, and improved sensory properties (Kenari & Razavi, 2022; Wang et al., 2020).

Antioxidant properties of plants are effective in delaying oxidation and rancidity in fats and oils and they have similar activity as chemically synthetic antioxidants (Aly et al., 2021; Wang et al., 2020). Natural extracts from different herbs, such as *Heracleum persicum* (Kenari et al., 2020), *Fumaria parviflora* L. (Razavi & Kenari, 2021), sesame (Esmaeilzadeh Kenari & Razavi, 2022), and *Rosmarinus officinalis* L. (Jafari et al., 2022), are stable for oxidation which is related to the presence of natural phenolic compounds.

Sage (*Salvia officinalis* L.), an evergreen shrub, belongs to the mint family (*Labiatae*). It is known for its aroma, flavor, and taste. Sage contains a wide array of bioactive compounds like phenolics, terpenoids, and organic acids that have shown antioxidant, antimicrobial, anticancer, and anti‐inflammatory activities (El‐Sayed & Youssef, 2019; Naziruddin et al., 2022). The extraction of bioactive compounds from plant materials with conventional methods such as maceration, shaker, and hydro‐distillation is laborious due to long extraction time, low efficiency, and hazardous solvents (Wrona et al., 2017). Ultrasound‐assisted extraction (UAE) process is a potentially useful technique for the purification and isolation of bioactive compounds. The high‐intensity and high‐frequency sound waves and also their interaction with plant materials distinguish UAE from the conventional methods (Sadat et al., 2021).

The efficiency of plant extracts pertains to biological activities and physicochemical properties. Low stability and water solubility, and the unpleasant taste of plant extract limit their application in food formulation. Encapsulation is a technology for maintaining the biological activities, control release, and bioavailability of bioactive compounds from plant materials which allow their application in different food formulations and preserving their functional properties (Reddy et al., 2022). It also enclosed bioactive compounds from light, oxygen, pH, water, and other adverse conditions (Jamshidi et al., 2020). A range of food‐grade biopolymers is used to create nanoparticles such as polysaccharides, proteins, and a combination of them (Razavi et al., 2021). Seed gums are new and plentiful polysaccharides. The *Lepidium perfoliatum* seed, which is known as Qodumeh Shahri in Iran, produces a high amount of mucilage. It can immobilize and bind a lot of water, and increase the viscosity of foods (Jamshidi et al., 2020). Whey protein isolate is obtained during the production of cheese or casein and it is a by‐product of the dairy industry which is widely used in the food industry because of its functional properties, emulsification, gelatinization, film formation, and solubility in water (Tavares & Noreña, 2019).

Considering that sunflower oil is sensitive to oxidation like other vegetable oils, it is necessary to increase its shelf life by adding natural antioxidants as safe preservatives. The use of extract encapsulation controls the release of antioxidant compounds from the extract during the storage. To the best of our knowledge, studies carried out so far have predominantly focused on using free extracts to increase the shelf life of vegetable oils. Also, no research has been published about the antioxidant activity of the encapsulated sage extract in whey protein isolate and Qodumeh Shahri (*Lepidium perfoliatum*) seed gum in sunflower oil. Therefore, the present study aimed to evaluate (1) the antioxidant activity of the sage extract, (2) the effect of coating material on the properties of nanocapsules, and (3) the effect of free and nanoencapsulated extract on the extension of oxidative stability of sunflower oil during the accelerated thermal condition.

## 2 MATERIAL AND METHODS

### 2.1 Material

The common sage was collected from the local field area near Sari (Mazandaran, Iran) in the summer of 2021. Sunflower oil without antioxidant was purchased from North Agro‐industrial Oil Company. All solvents and chemicals were purchased from Sigma‐Aldrich Company (Sigma). Qodumeh shahri seed gum was purchased from Reyhan gum parsian.

### 2.2 Methods

#### 2.2.1 Preparation of sage leaf extract

The leaves of sage were dried immediately after harvesting in a shady place for 1 week and the moisture content was below 10%. The dried sage leaves were ground into powder using a mechanical grinder (Habi, Pars‐Khazar). The powder was sieved using a 200‐μm sieve to remove any large pieces. To prepare sage leaf extract, 50 g of sage leaves was mixed with 250 ml of ethanol: water (70:30) solvent. The extraction was done using a ultrasonic bath (6.5l200 H, Dakshin, India) at 35°C for 30 min at a frequency of 35 kHz. The mixture was filtered using Whatman paper No. 1. Then, the solvent was evaporated using a rotary evaporator (RE 120) at 35°C and the final extract was kept at −18°C (Razavi & Kenari, 2021).

#### 2.2.2 Total phenolic content of sage leaf extract

The total phenolic content (TPC) of sage leaf extract was calculated according to the method reported by Doymaz and Karasu (2018). Initially, 2.5 ml of Folin–Ciocalteu phenol reagent (0.2 N) was added to 0.5 ml of extract and mixed with 2 ml of Na_2_CO_3_ (7.5%). This mixture was kept for 20 min at room temperature in a dark place. After incubation, the absorbance was recorded at 760 nm using a ultraviolet–vis spectrophotometer (Cintra 6, GBS Scientific). The total phenolic content was expressed as a gallic acid calibration curve (Doymaz & Karasu, 2018).

#### 2.2.3 Determination of antioxidant activity

The antioxidant activity of the extract was determined using 2,2‐diphenyl‐1‐picrylhydrazyl radical scavenging method (DPPH) and ferric reduction antioxidant power (FRAP). Briefly, 0.1 ml of extract and 4.9 ml of DPPH solution (0.1 mM in ethanol) were mixed toughly and held at 25°C for 30 min. Then, the absorbance was recorded at 517 nm and 3 ml of freshly prepared FRAP solution including FeCl_3_.6H_2_O (0.02 M in water), TPTZ (0.01 M dissolved in 0.04 M HCL), and acetate buffer (0.3 M, pH = 3.6) at the ratio 1:1:10 was mixed with 10 μl of extract. An increase in absorbance was recorded after 30 min at 593 nm. Antioxidant activity was expressed as mmol/g Trolox (Doymaz & Karasu, 2018).

#### 2.2.4 Sage leaf extract encapsulation

Whey protein isolate and qodumeh shahri seed gum solution at different ratios (1:0, 1:1, and 0:1) were used as coating materials. Initially, 0.05 g of coating powders was dispersed in deionized water at 30°C and after cooling, mixed overnight to enhance hydration. Then, 10 ml of sage extract was combined with 40 ml of tween 80 and 50 ml of sunflower oil during homogenizing with a magnetic stirrer at 100 rpm for 15 min. After that, the formed emulsion was homogenized again using Ultra‐Turrax homogenizer (IKA Labortechnik) at 15,000 rpm for 10 min followed by adding coating solution to nanoemulsion at a 5:1 ratio (Jafari et al., 2022).

#### 2.2.5 Properties of encapsulated sage extract

Nanoemulsions were dried using a freeze dryer (SP Scientific) at −50°C and 0.017 mPa for 48 h. The particle size, polydispersity index, and zeta potential of nanoemulsions were measured using a master‐sizer light scattering (Malvern Instrument Ltd.). To evaluate the encapsulation efficiency (EE) of sage extract, 200 mg of different nanoemulsions was mixed with hexane: water: methanol (50:42:8 v/v/v) to destroy the coat of nanocapsules. The surface phenolic content (SPC) and the total phenolic content (TPC) were measured. The EE was calculated using Equation 1:

(1) $$\mathrm{EE}\;\left(\%\right)=\left[\frac{\left(\mathrm{TPC}–\mathrm{SPC}\right)}{\mathrm{TPC}}\right]\times 100$$

The surface morphology of nanoemulsions was examined by SEM (Malvern Instrument Ltd.). Different nanoemulsions were fixed onto double‐sided adhesive carbon tabs mounted on SEM stubs, coated with gold (Kenari et al., 2020).

#### 2.2.6 Release rate of phenolic compounds

The release rate of phenolic compounds was measured according to the method described by Esmaeilzadeh Kenari et al. (2020). Initially, 20 g of different nanoparticles was poured into separate bottles and kept in an incubator at 60°C for 24 days. Then, 5 ml of phosphate buffer was mixed with 5 g of nanoparticles and centrifuged for 90 min at 1500 ***g*** and room temperature. The TPC of the lower phase was determined. The release rate was calculated using Equation 2 (Kenari et al., 2020):

$$\text{Release rate}\;\left(\%\right)=100–\left(100\times \frac{\text{Encapsulated}\;\mathrm{TPC}\;\text{in the outer phase}}{\text{Encapsulated}\;\mathrm{TPC}\;\text{in the inner phase}}\;\right)$$

#### 2.2.7 Oil storage and tests

Free (FREE) and nanoencapsulated sage extract in different seed gum (SGUM), whey protein isolate (WHEY), and complex coatings (COMP) were added to sunflower oil at 250 ppm. Synthetic TBHQ antioxidant (TBHQ) was employed at 100 ppm of concentration to compare the efficiency of sage extract. A control (CONT) sample without antioxidant and other samples were placed in separate bottles and kept in an incubator at 60°C for 24 days. Oil samples were removed periodically every 0, 4, 8, 12, 16, 20, and 24 days for analysis. The release rate of phenolic compounds (Jafari et al., 2022), peroxide value (PV), thiobarbituric acid value (TBA), conjugated dienes (CD) (AOCS, 2009), oxidative stability index (OSI) (Farahmandfar et al., 2018), and color index (CI) were determined (Kenari et al., 2020) every 4 days.

### 2.3 Statistical analysis

All experiments were performed in triplicate. Experimental data were analyzed using SPSS software (Statistical Program for Social Sciences) version 22. Significant differences (*p* < .05) were calculated using Duncan's multiple tests.

## 3 RESULTS AND DISCUSSION

### 3.1 Total phenolic content of sage extract

The total phenolic content (TPC) of sage extract was 31.12 mg GA/g. Nutrizio et al. (2020) explored high‐voltage electrical discharge and conventional method for extracting bioactive compounds from sage. They reported 19.67 and 42.13 mg GAE/g for conventional and electrical discharge extraction, respectively (Nutrizio et al., 2020). The TPC of aqueous extract of sage obtained by hot water extraction was 89.65 mg CA/g DW (Kontogianni et al., 2022). A value of 73.7 mg CA/g DW was reported by Kontogianni et al., 2013 for sage extract (Kontogianni et al., 2013). The difference in TPC may be attributed to the extraction time and temperature, type of solvent, extraction method, and variety of sage plants. Hamrouni‐Sellami et al. (2013) measured the effect of different drying temperatures on TPC of sage extract. They reported TPC from 0.4 to 2.5 mg GAE/g DW (Hamrouni‐Sellami et al., 2013).

### 3.2 Antioxidant activity of sage extract

**FIGURE 1:**
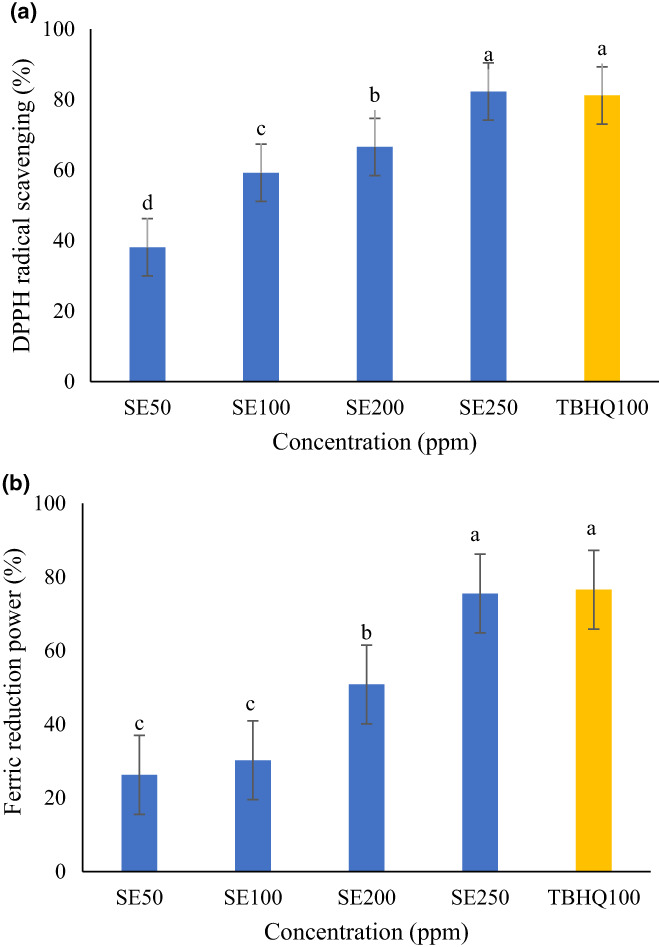
Antioxidant activity of sage extract. (a) DPPH radical scavenging activity, (b) ferric reduction antioxidant power

The antioxidant activity of sage extract was determined by the DPPH radical scavenging and FRAP assay. Figure 1a,b presents the antioxidant properties of different concentrations of sage extract. The antioxidant activity of extract was increased by increasing extract concentration. A statistically significant difference was observed between samples in the DPPH method. In the FRAP assay, the concentration of 50 and 100 ppm of extract has no statistically significant difference. Notably, the sage extract at 250 ppm had higher antioxidant activity than TBHQ in both DPPH and FRAP methods. Hamrouni‐Sellami et al. (2013) reported higher antioxidant activity of sage extract than BHA, HT, and ascorbic acid in DPPH, FRAP, and β‐carotene assay (Hamrouni‐Sellami et al., 2013) which is in line with the results of our study. The finding of the present study demonstrated that 250 ppm of sage extract could exhibit antioxidant activity equal to synthetic THQ antioxidant. Bigi et al. (2021) incorporated the sage extract into biopolymeric chitosan/hydroxypropyl methylcellulose coating and reported antioxidant activity due to the presence of bioactive phenolic compounds such as phenolic and flavonoids (Bigi et al., 2021). The antioxidant activity of sage extract related to presence of carnosol, rosmarinic acid, rosmanol, quinic acid, and carnosic acid (Generalić et al., 2012; Kontogianni et al., 2013; Oudjedi et al., 2019). Kontogianni et al., 2013 reported antioxidant activity for sage extract in both DPPH and FRAP methods which was IC_50_ = 27.41 μg DW/ml, and 536.81 mg Trolox/DW (Kontogianni et al., 2013). The antioxidant activity of sage extract obtained by electrical discharge, conventional method, and microwave also was reported by other researchers (Generalić et al., 2012; Hamrouni‐Sellami et al., 2013; Nutrizio et al., 2020). Similarly, literature reported a significant increase in both DPPH and FRAP antioxidant activity by an increase in the TPC of extract (Esmaeilzadeh Kenari & Razavi, 2022; Kenari et al., 2020; Razavi & Kenari, 2021).

### 3.3 Properties of nanocapsules

The results of the particle size of different nanocapsules are shown in Table 1. All nanocapsules showed a size below 270 nm and a statistically significant difference was observed. The pressure of ultra‐turrax beside sonication energy caused nanosize of particles (Razavi et al., 2020). PDI is among the most important characteristic of nanocarrier systems. PDI of all samples was below 0.300 which indicates the normal distribution of particle size. The zeta potential is helpful to determine the net charge of nanocapsules. Zeta potential of all nanocapsules was negative. It is because of negative nature of whey protein isolate and anionic compounds in seed gum. Tavares and Noreña (2019) reported a negative charge for encapsulated extract in whey protein isolate and chitosan which is due to the negative charge of whey protein isolate (Tavares & Noreña, 2019). The lower zeta potential was observed in nanocapsule prepared using complex coating which attributed to intensifying the negative charge. EE of extract ranged from 61.54% to 74.77%. The higher and lower EE was observed in nanocapsule prepared by seed gum followed by whey protein isolate, respectively. The EE higher than 50% was also reported by other researchers (Hosseinialhashemi et al., 2020; Razavi et al., 2020; Rezaei Savadkouhi et al., 2020).

**TABLE 1:** Particle size, PDI, zeta potential, and encapsulation efficiency of nanocapsules

Sample	Particle size (nm)	PDI	Zeta potential (mV)	EE (%)
SGUM	270.0 ± 6.7a	0.288 ± 0.02c	−35.2 ± 2.1b	74.77 ± 4.2a
WHEY	255.3 ± 5.4b	0.294 ± 0.04b	−24.17 ± 1.8a	61.54 ± 4.0c
COMP	217.4 ± 6.2c	0.300 ± 0.01a	−41.36 ± 3.6c	68.16 ± 3.5b

*Note*: Different letters indicate statistically significant differences (*p* < .05) between samples.

### 3.4 Morphology of nanocapsules

**FIGURE 2:**
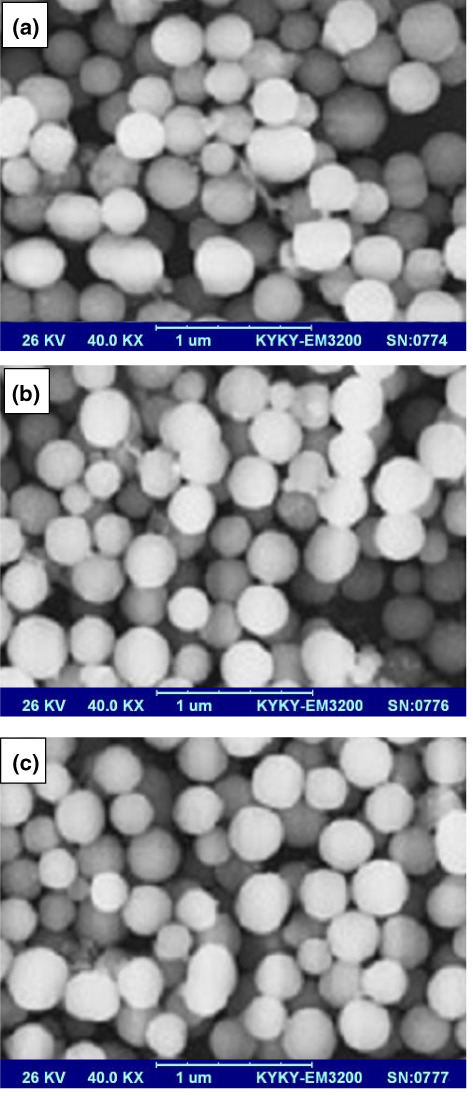
SEM images of nanoencapsulated sage extract in different coatings. (a) Whey protein isolate, (b) seed gum, and (c) complex of protein and gum

The morphological structure of nanocapsules depends on the interactions between the coating components, which affect the final physiochemical properties. The surface morphology of nanocapsules is presented in Figure 2. The surface of all nanocapsules was smooth and did not show cracks, pores, and bubbles. Figure 2c indicates the formation of high compatibility between gum and protein to form wall coating. These surface morphology images confirmed that the sage extract was well encapsulated into the polymer matrix (Esmaeilzadeh Kenari & Razavi, 2022). A similar result was observed in a nanocapsule of Iranian golpar (Kenari et al., 2020), *Fumaria parviflora* (Razavi & Kenari, 2021), rosemary leave (Jafari et al., 2022), and sesame seed extract (Esmaeilzadeh Kenari & Razavi, 2022).

### 3.5 Release rate of phenolic compounds

The gradual release rate of phenolic compounds was observed in all samples (Table 2) and differences were significant. There is a positive correlation between size diameter and release rate of phenolic compounds from nanoparticles. This result is in line with the reports of other researchers on the gradual release of phenolic compounds from extracts of Iranian golpar (Kenari et al., 2020), rosemary leaf (Jafari et al., 2022), olive leaf (Mohammadi et al., 2016), and *Ferula persica* into soybean oil (Estakhr et al., 2020).

**TABLE 2:** Release rate of phenolic compounds from nanocapsules

Sample	0	4	8	12	16	20	24
SGUM	5.17 ± 1.1a	10.21 ± 1.2a	16.48 ± 2.0a	25.76 ± 3.5a	39.91 ± 4.2a	51.42 ± 5.1a	66.70 ± 5.3a
WHEY	5.02 ± 0.9b	8.22 ± 1.0b	11.35 ± 2.4b	20.76 ± 1.2b	28.70 ± 3.2b	34.91 ± 4.8b	48.52 ± 2.1b
COMP	4.81 ± 0.8c	7.45 ± 1.1c	10.36 ± 1.5c	17.08 ± 2.5c	22.19 ± 2.7c	30.25 ± 2.7c	43.22 ± 3.5c

*Note*: Different letters indicate statistically significant differences (*p* < .05) between samples.

### 3.6 Oil oxidation

**FIGURE 3:**
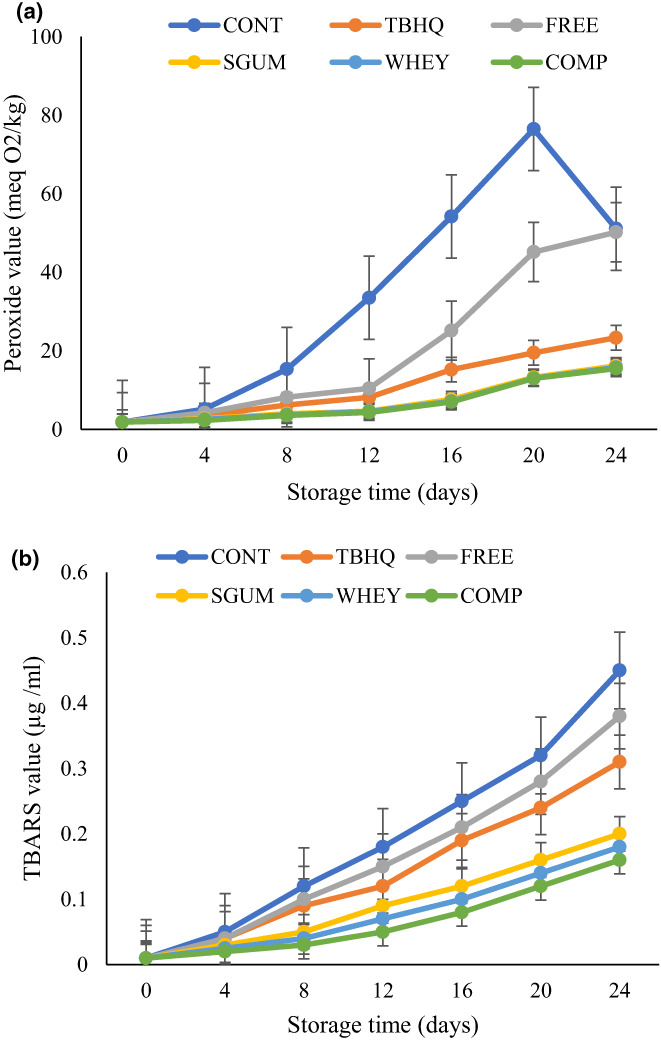
Oxidation of sunflower oil during storage. (a) Peroxide value, and (b) thiobarbituric acid value

Oils with high degree of unsaturation are prone to autooxidation. The simplest test for evaluating the oil autooxidation is PV and TBA. Figure 3a shows the values of PV for each sample in relation to the days of storage at 60°C. In all samples, a continuous increase in PV was observed over time. In the control sample after primary oxidation and maximum PV, a decrease in PV was observed which indicates the stage where the rate of decomposition of peroxide is higher than the rate of peroxide formation. The PV of all samples at the initial time was 1.86 meq/kg. Therefore, the rate of oil oxidation during storage depends on the type of antioxidants being added. The control sample exhibited the highest level of peroxides during storage (76.48 meq/kg) and at 20 days of storage, a decrease in PV was observed. During the storage, sunflower oil containing nanoencapsulated sage extract showed lower PV than oil containing the free sage extract. In other words, the nanoencapsulated extract was more effective to delay the oxidation process than the free extract during the first stage. A similar result was observed by Royshanpour et al. (2020) who reported lower PV in soybean oil enriched with nanoencapsulated *M. piperita* than in free extract (Royshanpour et al., 2020). The control sample exhibited higher PV followed by FREE, TBHQ, SGUM, WHEY, and COMP. In a study conducted by Dauber et al. (2022), the antioxidant activity of olive leaf extract in canola oil was measured. A higher PV was observed in the sample without antioxidant and the oil‐containing extract exhibited lower PV due to the presence of phenolic compounds in the extract (Dauber et al., 2022). Hosseinialhashemi et al. (2020) stated the higher efficiency of encapsulated *Pistacia khinjuk* extract than TBHQ on extension of sunflower oil stability (Hosseinialhashemi et al., 2020).

TBA value gives a measure of oil oxidation development, in terms of secondary products of oil oxidation. The results of the TBA value of different samples in Figure 3b show an increasing rate in TBA of all samples. The control sample exhibited a higher TBA value followed by FREE, TBHQ, SGUM, WHEY, and COMP. Aleena et al. (2020) measured the oxidative stability of sunflower oil in high‐temperature cooking. Their results showed an increase in lipid oxidation during heating (Aleena et al., 2020) which is in accordance with the results of the present study. Binsi et al. (2017) increased the oxidative stability of fish oil using sage extract and oil encapsulation. Their results revealed that sage could extend the shelf life of fish oil according to the PV and TBA (Binsi et al., 2017). An increasing trend in the TBA value of plant oil was also reported for soybean oil containing free and nanoencapsulated olive leaf extract (Taghvaei et al., 2014), and potato skin extract in soybean oil (Tavakoli et al., 2021).

**FIGURE 4:**
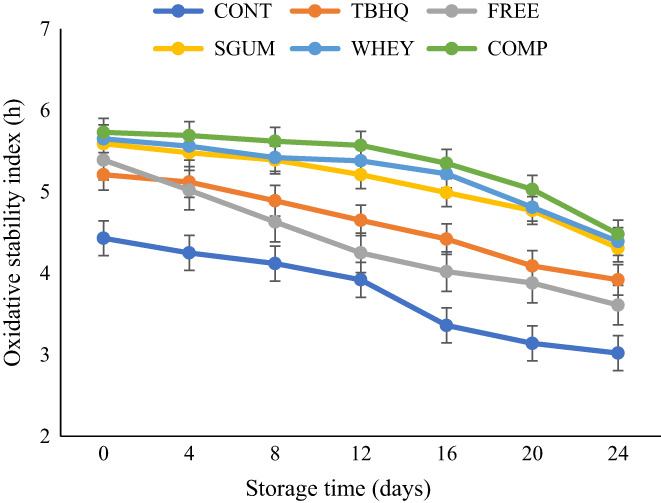
Oxidative stability of sunflower oil during storage

The oxidative stability index (OSI) is defined as the point of maximum variation of oil oxidation rate. The results of oxidative stability of sunflower oil samples which were performed at 110°Care illustrated in Figure 4. The OSI of all samples decreased over time. Continued decrease in OSI with an increase in storage time was reported for sunflower oil with pussy willow extract (Sayyari & Farahmandfar, 2017). Control sample showed a lower OSI. Also, oil samples containing encapsulated sage extract exhibited higher OSI, which is related to the antioxidant activity of sage extract. In a study conducted by Upadhyay and Mishra (2015), the sage extract was found to have protective effect on oxidative stability of sunflower oil (Upadhyay & Mishra, 2015). Taghvaei et al. (2014) found that the thermal stability of soybean oil containing olive leaf extract in both free and encapsulated forms is higher than blank oil. However, oil containing encapsulated extract showed higher OSI (Taghvaei et al., 2014) which is in accordance with the results of the present study.

**FIGURE 5:**
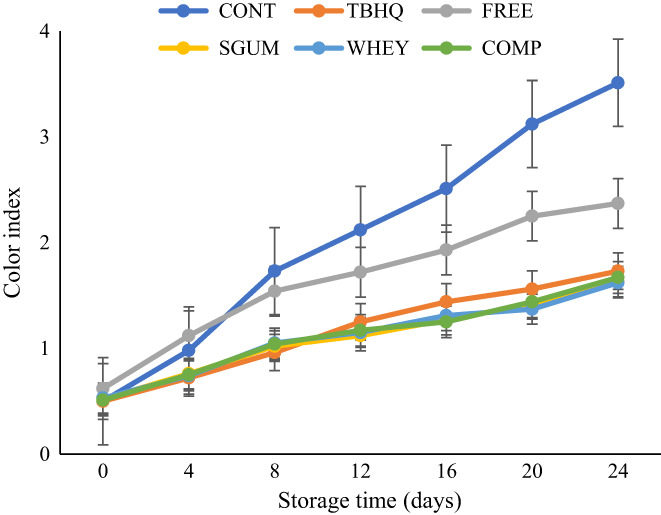
Color index of sunflower oil during storage

Color is considered a vital indicator contributing to the quality of edible oils and consumer preference. The results of color index of different oil samples are presented in Figure 5. The color index of all samples increased over time. At the end of storage time, control sample showed a higher color index. During storage time under thermal conditions, the yellow color of sunflower oil turns dark which is related to the oil oxidation process. Therefore, the oil samples containing TBHA and encapsulated extract showed a lower color index. The decomposition of secondary metabolites to smaller compounds and the formation of polymeric triglycerides was much more in control sample. The colorful compounds present in the sage extract cause higher color index of oil than the control sample on days 0 and 4 of storage. The encapsulation process led to the placement of extract's compounds inside the coating and decreased color index. These results are in accordance with the results reported by Kenari et al. (2020), who reported higher color index in soybean oil without antioxidant followed by oil containing TBHQ, nanoencapsulated Iranian golpar extract, and free extract (Kenari et al., 2020). Similar results were reported by Salami et al. (2020) for lower color index of canola oil containing TBHQ than oil with pumpkin peel extract (Salami et al., 2020).

**FIGURE 6:**
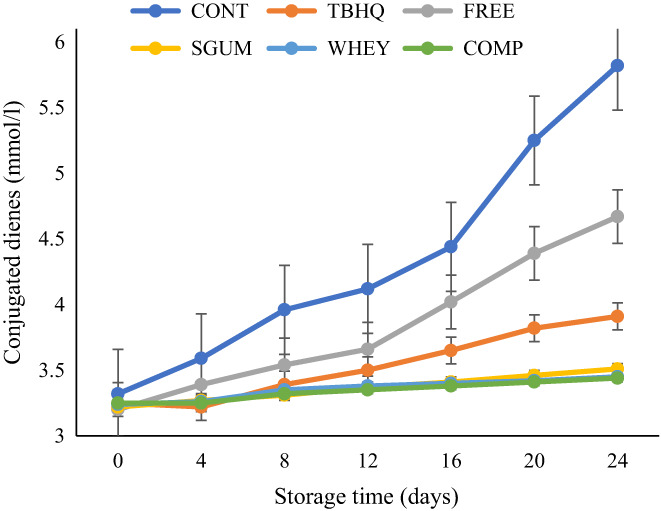
Conjugated dienes of sunflower oil during storage

Another indicator for evaluating oil oxidation is conjugated dienes (CD). These compounds are formed by the rearrangement of double bounds of hydroperoxides during oil oxidation. The results of CD of different oil samples are shown in Figure 6. A continuous increase in CD value was observed in line with the lengthening of the storage period for all samples. Similar to PV, the oil samples containing nanoencapsulated sage extract showed lower CD value. The CD value represents the primary degradation products of oil and confirms the PV content of oil samples. Talón et al. (2019) reported lower CD value in sunflower oil containing encapsulated eugenol (Talón et al., 2019). An increasing trend in CD value of plant oil during thermal processing and storage time, and also low CD value of oil containing plant extracts previously reported by other publications (Kenari et al., 2020; Maghsoudlou et al., 2017; Salami et al., 2020; Talón et al., 2019).

## 4 CONCLUSION

In this study, the antioxidant effect of free and nanoencapsulated sage extract was compared to TBHQ synthetic antioxidant. All coating materials could extend the antioxidant properties of sage extract by controlling gradual release of phenolic compounds, and protecting phenolic compounds from environmental stresses. According to the results of oxidation parameters of oil, the use of complex coating of whey protein isolate and qodumeh shahri seed gum was suggested to encapsulate sage seed gum as a natural antioxidant to extend the shelf life and oxidative stability of sunflower oil.

## CONFLICT OF INTEREST

The authors declare no conflict of interest.

## ETHICS STATEMENT

The study does not involve any human or animal testing.

## Data Availability

Research data are not shared.
